# Norepinephrine Inhibits the Proliferation of Human Bone Marrow-Derived Mesenchymal Stem Cells via β2-Adrenoceptor-Mediated ERK1/2 and PKA Phosphorylation

**DOI:** 10.3390/ijms21113924

**Published:** 2020-05-30

**Authors:** Jessica Hedderich, Karima El Bagdadi, Peter Angele, Susanne Grässel, Andrea Meurer, Rainer H. Straub, Frank Zaucke, Zsuzsa Jenei-Lanzl

**Affiliations:** 1 Dr. Rolf M. Schwiete Research Unit for Osteoarthritis, Orthopedic University Hospital Friedrichsheim, 60528 Frankfurt/Main, Germany; jessica.hedderich@friedrichsheim.de (J.H.); karima.elbagdadi@friedrichsheim.de (K.E.B.); andrea.meurer@friedrichsheim.de (A.M.); frank.zaucke@friedrichsheim.de (F.Z.); 2 Laboratory of Experimental Trauma Surgery, Department of Trauma Surgery, University Hospital Regensburg, 93053 Regensburg, Germany; angele@sporthopaedicum.de; 3 Department of Orthopedic Surgery, Experimental Orthopedics, Centre for Medical Biotechnology (ZMB), University of Regensburg, 93053 Regensburg, Germany; susanne.graessel@ukr.de; 4 Laboratory of Experimental Rheumatology and Neuroendocrine Immunology, Department of Internal Medicine I, University Hospital Regensburg, 93053 Regensburg, Germany; rainer.straub@ukr.de

**Keywords:** bone marrow-derived mesenchymal stem cells (BMSCs), proliferation, regeneration, sympathicus, norepinephrine

## Abstract

Bone marrow-derived mesenchymal stem cells (BMSCs) represent an alternative to chondrocytes to support cartilage regeneration in osteoarthritis (OA). The sympathetic neurotransmitter norepinephrine (NE) has been shown to inhibit their chondrogenic potential; however, their proliferation capacity under NE influence has not been studied yet. Therefore, we used BMSCs obtained from trauma and OA donors and compared the expression of adrenergic receptors (AR). Then, BMSCs from both donor groups were treated with NE, as well as with combinations of NE and α1-, α2- or β1/2-AR antagonists (doxazosin, yohimbine or propranolol). Activation of AR-coupled signaling was investigated by analyzing ERK1/2 and protein kinase A (PKA) phosphorylation. A similar but not identical subset of ARs was expressed in trauma (α2B-, α2C- and β2-AR) and OA BMSCs (α2A-, α2B-, and β2-AR). NE in high concentrations inhibited the proliferation of both trauma and OA BMCSs significantly. NE in low concentrations did not influence proliferation. ERK1/2 as well as PKA were activated after NE treatment in both BMSC types. These effects were abolished only by propranolol. Our results demonstrate that NE inhibits the proliferation and accordingly lowers the regenerative capacity of human BMSCs likely via β2-AR-mediated ERK1/2 and PKA phosphorylation. Therefore, targeting β2-AR-signaling might provide novel OA therapeutic options.

## 1. Introduction

Mesenchymal stem cells (MSCs) are multipotent progenitor cells with high proliferative activity [1,2]. Their existence in joint tissues such as synovium, bone marrow, and articular cartilage as well as their role in cartilage regeneration has been confirmed in previous studies [3,4,5]. Especially bone marrow-derived MSCs (BMSCs) have been described to migrate to articular cartilage defects and to either differentiate into chondrocytes or to influence the self-regeneration capacity of cartilage by acting as trophic mediators [6,7]. Furthermore, recent studies used isolated BMSCs in clinical applications for cartilage repair in knee trauma or osteoarthritis (OA) patients; however, the success of intra-articular BMSC injection/application on articular cartilage repair was very limited [8]. The regenerative potential of BMSCs is directly proportional to the number of available BMSCs and accordingly dependent on their proliferation capacity [9]. The proliferation capacity of BMSCs is affected by numerous microenvironmental factors such as tissue origin, growth factors, oxygen concentration, presence of inflammatory mediators or hormones [9,10,11]. The bone marrow is innervated by sympathetic nerve fibers [12] and thus, the sympathetic nervous system may influence BMSCs proliferation. Sympathetic nerve fibers express tyrosine hydroxylase (TH), which is the key enzyme for the biosynthesis of catecholamines, such as e.g., norepinephrine (NE), one of the major peripheral sympathetic neurotransmitters. NE is indeed present in the bone marrow in physiological concentrations (up to 10^−9^ M) [12]. In addition, the synovial fluid of knee trauma and OA patients contains relevant NE concentrations, namely 10^−9^ M to 10^−7^ M, suggesting that BMSC proliferation might also be affected after migration from the bone marrow to the damaged cartilage area [13]. Furthermore, NE concentrations up to 10^−5^ M were measured locally at sympathetic nerve fiber terminals immediately after nerve activation and neurotransmitter release. Thus, in sympathetically innervated tissues, such as the bone marrow or the synovium and the synovial fluid, NE can be present in a concentration range from 10^−9^ to 10^−5^ M [14]. It was demonstrated in earlier studies that BMSCs express α- and β-adrenergic receptors (AR) belonging to the G-protein coupled receptors [13,15,16]. NE receptor binding affinity depends on its concentration [14]: At low concentrations (≤10^−7^ M) NE binds preferentially to α-ARs leading to decreasing cAMP concentrations and subsequently to inhibition of protein kinase A (PKA) [17]. At high NE concentrations (≥10^−6^ M), β-ARs are primarily activated resulting in cAMP increase and PKA activation (Gαs signaling). In addition, α- and β-ARs activation alternatively leads to extracellular signal-regulated kinases 1/2 (ERK1/2) pathway activation [18,19]. Previous studies demonstrated that NE influences the function of various musculoskeletal cell types: NE decreases the proliferation of human chondrocytes via β-AR signaling while low concentrations lead to increased proliferation and apoptosis rates via α1-AR signaling [20]. Similar effects of NE on the proliferation of human osteoblasts have been demonstrated in previous studies [21]. Furthermore, NE inhibits differentiation of murine chondrocytes via β2-AR leading to increased ERK1/2 phosphorylation and PKA activation and similarly [22], the chondrogenic differentiation potential of human BMSCs is reduced by treatment with NE via β2-AR-activation [13]. Nevertheless, no studies have been performed yet investigating the effects of NE on the proliferation capacity of human BMSCs. Therefore, we investigated if and how NE influences the proliferation of human BMSCs derived from trauma and knee OA patients. In addition, we further aimed to identify the involved ARs and AR-dependent signaling pathways. These findings will help to understand why endogenous BMSC-dependent repair processes or current clinical BMSCs applications often fail and might provide new therapeutic options by application of specific AR modulating drugs.

## 2. Results

### 2.1. AR and TH Gene Expression Profile of BMSCs

**Figure 1:**
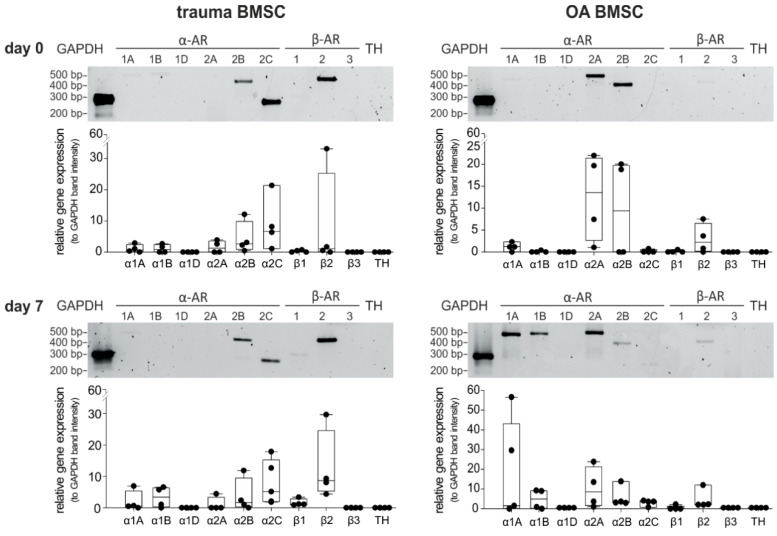
AR and TH gene expression profile of trauma and OA BMSCs. Gene expression of different AR subtypes and TH in untreated BMSCs isolated from trauma or OA donors in monolayer culture at day 0 and after 7 days (representative PCR gel image of one donor) and average score of gene expression relative to the expression of Glycerinaldehyd-3-phosphat-Dehydrogenase (GAPDH, *n* = 4, mean ± standard deviation). Data are shown as box plots where each box represents the 25th to 75th percentiles. The lines inside the boxes represent the median. Lines outside the boxes represent the 10th to 90th percentiles. Each black dot represents one individual donor.

Because expression of diverse subtypes of ARs is the precondition for potential NE-mediated effects, the gene expression profile of ARs in trauma and OA BMSCs kept under physioxic culture conditions (2% O_2_) was investigated by RT-PCR (mRNA levels). To recognize autocrine effects, gene expression of TH was also analyzed. Trauma and OA BMSCs expressed distinct subtypes of ARs on day 0 of monolayer culture. In trauma BMSCs, α2B-, α2C- and β2-AR were strongly expressed (Figure 1). In contrast, OA BMSCs showed low expression of β2-AR, while expression of α2A- and α2B-AR was strong. Neither trauma nor OA BMSCs expressed the receptors α1A, α1B-, α1D-, β1-, β3-AR or TH to a detectable amount on day 0. We also analyzed possible changes in the AR and TH gene expression profile after 7 days in monolayer culture without any treatment. We compared the relative gene expression data for each receptor subtype between day 0 and day 7. In addition, we compared the gene expression data for each receptor subtype with respect to age or gender for each time point. The gene expression profile of trauma and OA BMSCs showed no significant differences after 7 days in monolayer culture. No age or gender dependent differences were detected.

### 2.2. Proliferation Capacity of Trauma and OA BMSCs

**Figure 2:**
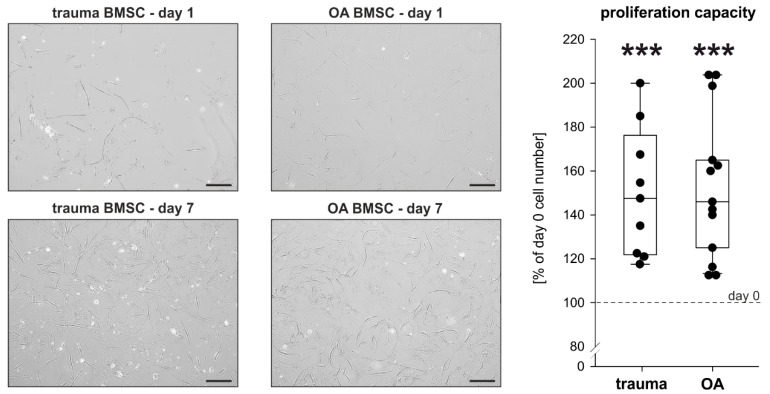
Proliferation capacity of trauma and OA BMSCs. Representative images at day 1 and day 7 and proliferation rates of trauma and OA BMSCs after 7 days in monolayer culture compared to day 0 (day 0 cell number: 200 000 cells; day 7 cell number: trauma BMSCs 300 181 ± 58 867 cells, mean proliferation rate 150.1%, *p* < 0.001; OA BMSCs 304 046 ± 60 148 cells, mean proliferation rate 152.0%, *p* < 0.001). Data are presented as percent of the initial cell count on day 0. Data are shown as box plots as explained in legend to Figure 1 (*n* = 9 for trauma BMSCs, *n* = 13 for OA BMSCs, bars 200 μm). Significant *p* values are presented as *** *p* < 0.001 to the initial controls at day 0.

To evaluate and compare the proliferation capacity of trauma and OA BMSCs without any treatment, proliferation rates after 7 days in monolayer culture under physioxic conditions were determined. Compared to the microscopic picture at day 1 (the time point when the seeded cells became adherent) an increase of cell numbers was clearly visible until day 7 in both trauma and OA BMSCs cultures (Figure 2). The cell numbers in both trauma and OA BMSC culture increased significantly from day 0 to day 7 (Figure 2). In comparison, no statistically significant changes of proliferation rates between trauma and OA BMSCs were detected (Figure 2).

### 2.3. Effect of NE on Proliferation of Trauma and OA BMSCs

**Figure 3:**
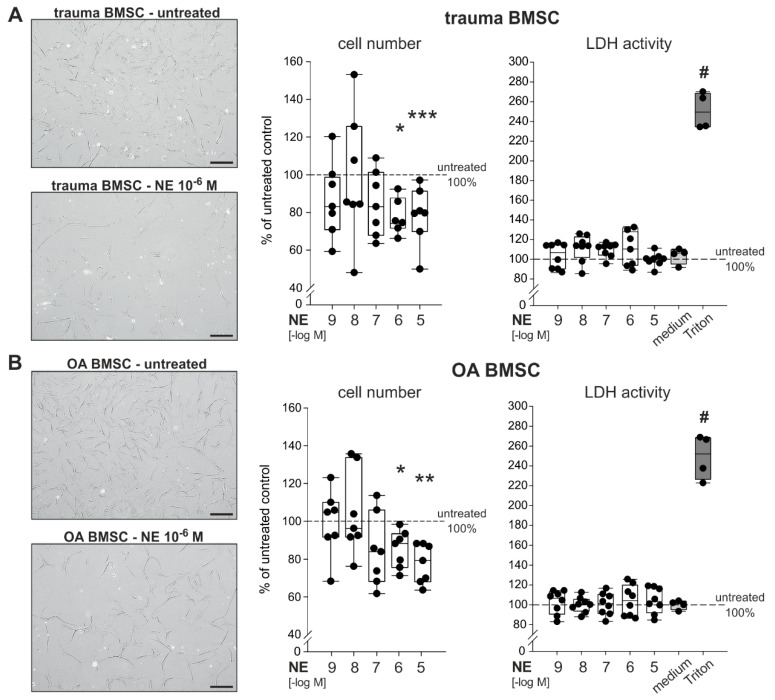
Effect of NE on the proliferation and viability of trauma and OA BMSCs (**A**) Representative microscopic image of trauma BMSCs in culture treated with NE (10^−6^ M) as well as vital cell count and LDH release of trauma BMSCs after 7 days of NE treatment (10^−9^ to 10^−5^ M) in monolayer culture (*p* < 0.001 for NE 10^−5^ M vs. untreated control, *p* = 0.014 for NE 10^−6^ M vs. untreated control; *n* = 6–7). (**B**) Representative microscopic image of OA BMSCs in culture treated with NE (10^−6^ M) as well as vital cell count and LDH release of OA BMSCs after 7 days NE of treatment (10^−9^ to 10^−5^ M) in monolayer culture (*p* = 0.002 for NE 10^−5^ M vs. untreated control, *p* = 0.014 for NE 10^−6^ M vs. untreated control; *n* = 7–8). Scale bars represent 200 µm. Data are presented as box plots as described in the legend to Figure 1 and as percent of the untreated control (control = 100%, dotted line). Significant *p* values are presented as * *p* ≤ 0.05, ** *p* < 0.01 or *** *p* < 0.001 to untreated control and ^#^ < 0.05 to untreated control and all treatment groups (in the case of LDH release).

BMSCs derived from trauma and OA patients were treated with NE in different concentrations (10^−9^ to 10^−5^ M) for 7 days in monolayer culture under physioxic conditions. After 7 days, BMSCs treated with high concentrations of NE (10^−5^ to 10^−6^ M) showed an apparent microscopically visible decrease of confluency. Treatment with NE led to a clear and dose-dependent reduction in cell numbers on day 7 compared to the untreated control group (Figure 3A,B). Especially high concentrations of NE (10^−5^ to 10^−6^ M) significantly reduced cell numbers in both trauma (Figure 3A) and OA (Figure 3B) cell cultures. NE in low concentrations (10^−9^ to 10^−7^ M) had no effect on cell numbers. Cell viability of trauma and OA BMSCs, measured by lactate dehydrogrenase (LDH) activity, was not affected by any treatment.

### 2.4. Changes in AR Gene Expression after Treatment with NE

**Figure 4:**
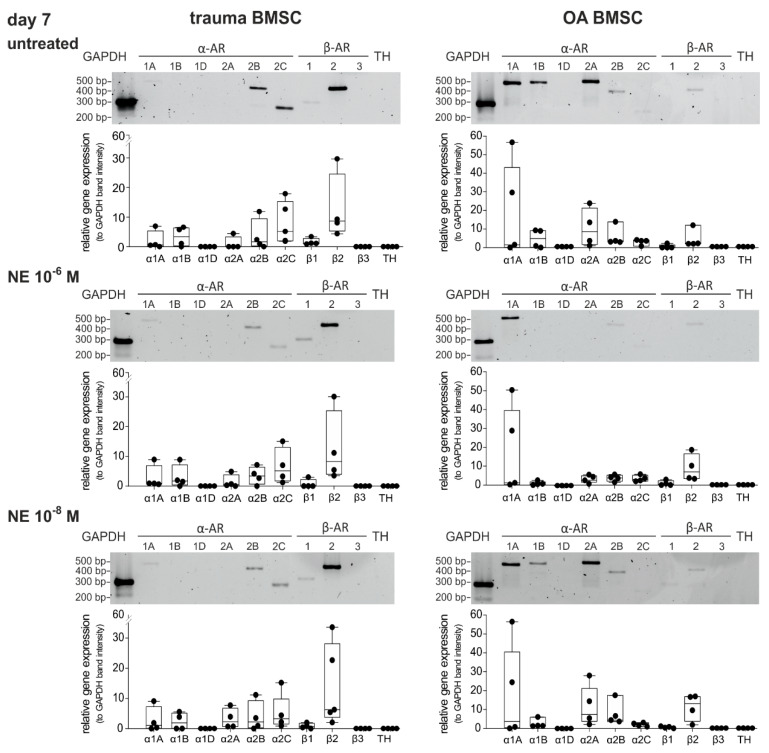
Changes in AR expression after NE treatment. Gene expression of different AR subtypes in untreated trauma and OA BMSCs in monolayer culture at day 7 and after treatment with NE 10^−6^ M and NE 10^−8^ M (representative image of one trauma and OA patient) and average score of gene expression (*n* = 4, mean ± standard deviation). Data are shown as box plots explained in legend to Figure 1.

We investigated if NE treatment over 7 days led to alterations in AR gene expression of trauma and OA BMSCs (Figure 4). In trauma BMSCs neither high nor low concentrations of NE had any effect on the AR expression profile compared to untreated controls (Figure 4). In OA BMSCs, the expression of α2A- and α2B-AR slightly decreased (not significant) after treatment with NE in high concentrations (10^−6^ M) in comparison to the untreated control group, while the expression of α1A-, α1B- and β2-AR did not change significantly. In contrast, treatment with NE in low concentrations (10^−8^ M) had no influence on the AR expression profile of OA BMSCs.

### 2.5. NE-Induced Intracellular Signaling

**Figure 5:**
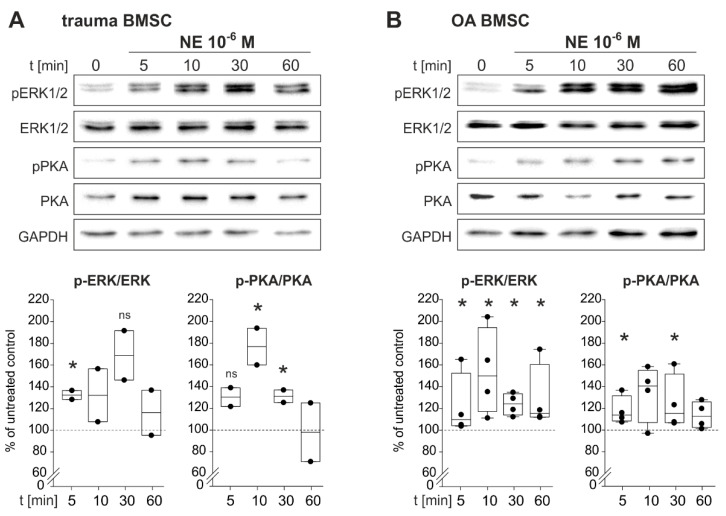
NE-mediated activation of intracellular signaling pathways in trauma and OA BMSCs. (**A**) Western Blot analysis of ERK1/2 as well as PKA phosphorylation in trauma BMSCs treated with NE 10^−6^ M for 5, 10, 30 and 60 min. (*n* = 2, representative blot of one trauma patient, ERK1/2: *p* = 0.045 for 5 min of NE stimulation vs. untreated control group, *p* = 0.07 not significant for 30 min of NE stimulation vs. untreated control group; PKA: *p* = 0.075 not significant for 5 min of NE stimulation vs. untreated control group, *p* = 0.042 for 10 min of NE stimulation vs. untreated control group, *p* = 0.045 for 30 min of NE stimulation vs. untreated control group). (**B**) Western Blot analysis of ERK1/2 as well as PKA phosphorylation in OA BMSCs treated with NE 10^−6^ M) for 5, 10, 30 and 60 min. (*n* = 4, representative blot of one OA patient, ERK1/2: *p* < 0.029 for 5 min of NE stimulation vs. untreated control group, *p* = 0.036 for 10 min, *p* = 0.018 for 30 min and *p* < 0.029 for 60 min; PKA: *p* = 0.029 for 10 min, *p* = 0.029 for 30 min). Data are shown as box plots explained in legend to Figure 1. Significant *p* values are presented as * *p* ≤ 0.05 to the untreated control, ns – not significant.

To identify NE-induced intracellular signaling pathways, BMSCs of trauma and OA patients were treated with NE (10^−6^ M) for 5, 10, 30 and 60 min. NE treatment resulted in an increase of ERK1/2 and PKA phosphorylation already after 5 min (Figure 5).

In trauma BMSCs, NE treatment resulted in a statistically significant increase of ERK1/2-phosphorylation already after 5 min. Maximal ERK1/2-phosphorylation was reached after 30 min and started to decline after 60 min of stimulation. An increase in PKA phosphorylation was already detected after 5 min of NE treatment. Maximal phosphorylation was reached after 10 min. After 30 min of stimulation, PKA activation declined and reached its initial level after 60 min (Figure 5A). In OA BMSCs, phosphorylation of ERK1/2 increased significantly after 5 min and ERK1/2 was maximally activated after 10 min. Activation remained constant up to 60 min of stimulation. PKA phosphorylation increased significantly already after 5 min. The maximum was reached after 10 min and remained constant up to 60 min of stimulation (Figure 5B).

### 2.6. Reversal of NE-Mediated Effects by β2-AR-Antagonist

**Figure 6:**
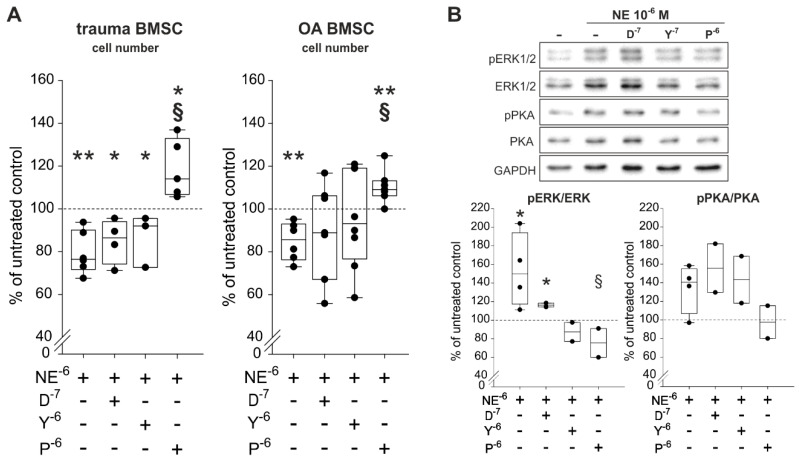
Proliferation and intracellular signaling in trauma and OA BMSCs treated with NE and AR antagonists. (**A**) Vital cell count of trauma and OA BMSCs treated with NE in combination with α1-, α2- or β2-AR antagonists for 7 days in monolayer culture (trauma BMSCs: *p* = 0.004 for NE 10^−6^ M vs. untreated control, *p* = 0.01 for NE 10^−6^ M + D 10^−7^ M vs. untreated control, *p* = 0.024 for NE 10^−6^ M + Y 10^−7^ M vs. untreated control, *p* = 0.036 for NE 10^−6^ M + P 10^−6^ M vs. untreated control, *p* < 0.001 for NE 10^−6^ M + *p* 10^−6^ M vs. stimulation with NE 10^−6^ M alone; OA BMSCs: *p* = 0.009 for NE 10^−6^ M vs. untreated control, *p* = 0.004 for NE 10^−6^ M + P 10^−6^ M vs. untreated control, *p* < 0.001 for NE 10^−6^ M + P 10^−6^ M vs. stimulation with NE 10^−6^ M alone; *n* = 3−7). (**B**) Western blot analysis of ERK1/2 and PKA phosphorylation in OA BMSCs stimulated with NE in combination with α1-, α2- or β2-AR antagonists for 10 min in monolayer culture (*n* = 2, representative blot of one OA BMSC donor; ERK1/2: *p* = 0.036 for NE 10^−6^ M vs. untreated control group, *p* = 0.016 for NE 10^−6^ M + D 10^−7^ M vs. untreated control group, *p* = 0.041 for NE 10^−6^ M + P 10^−6^ M vs. stimulation with NE alone; PKA: no significant differences for *n* = 2). Western Blot was only performed for OA BMSCs but based on the other results we would not expect any differences for trauma BMSCs. Data are presented as percent of the untreated control (=100%, broken line). Data are shown as box plots explained in legend to Figure 1. Significant *p* values are presented as * *p* ≤ 0.05 or ** *p* < 0.01 to the untreated control and as ^§^ *p* ≤ 0.05 to the NE-treated group. Abbreviations: D^−7^—doxazosin (α2-AR antagonist); Y^−6^—yohimbine (α2-AR antagonist); *p*^−6^—propranolol (β2-AR-antagonist)

To identify which ARs are involved in NE-mediated effects on proliferation and which are able to reverse the observed NE-mediated effects, trauma and OA BMSCs were treated with NE (10^−6^ M) in combination with specific AR antagonists for 7 days in monolayer culture under physioxic conditions. The α1-AR-antagonist doxazosin (D, 10^−7^ M), the α2-AR-antagonist yohimbine (Y, 10^−7^ M) as well as the β2-AR-antagonist propranolol (*p*, 10^−6^ M) were applied. Treatment of both trauma and OA BMSCs with NE in combination with propranlol led to a significant increased cell count compared to NE treatment alone (Figure 6A). Neither doxazosin nor yohimbine in combination with NE influenced the proliferation rates of trauma and OA BMSCs statistic significantly in comparison to treatment with NE alone (Figure 6A). Cell viability correlated with LDH activity, was not affected by any treatment compared to untreated control groups (Figure S1). For the sake of completeness, cells were also treated with agonists of the two major ARs, α2-AR-specific agonist UK14,304 (UK, 10^−8^ M) and the specific β2-AR-agonist formoterol (F, 10^−7^ M) were applied for 7 days. In both trauma and OA BMSCs, treatment with formeterol resulted in a significant reduction of the cell number to the same extent as NE in high concentrations (10^−5^ to 10^−6^ M) when compared to the untreated control group (Figure S2A). In contrast, UK14,304 had no influence on the proliferation of both trauma and OA BMSCs. Cell viability measured by LDH activity was not influenced by any treatment (Figure S2B).

Western Blot analysis of the ERK1/2 and the PKA pathways showed that only the specific β2-AR-antagonist propranolol was able to reverse NE-induced phosphorylation of ERK1/2 and PKA (Figure 6B). Doxazosin as well as yohimbine were not able to reverse the observed NE-mediated effects on ERK1/2 and PKA phosphorylation (Figure 6B).

## 3. Discussion

Bone marrow-resident stem cells are described as playing an important role in cartilage repair [23]. For this reason, diverse attempts have been performed to apply BMSCs intra-articularly in knee trauma or OA patients; however, the regeneration was not satisfying [8]. The extensive proliferation capacity of BMSCs, which is a prerequisite for regeneration processes, depends on several bone marrow microenvironmental factors [9]. Although the presence of sympathetic nerve fibers and the release of the major sympathetic neurotransmitter NE in the human bone marrow and in the synovial fluid is well known [12,13,24], no studies analyzing the effect of NE on the proliferation capacity of human BMSCs have been performed until now. Therefore, our study aimed to investigate the effect of NE on the proliferation of human BMSCs. We used BMSCs derived from trauma and knee OA patients, in order to compare the influence of tissue origin on their response to NE.

First, we determined the AR gene expression profile of human trauma and OA BMSCs because the expression of diverse subtypes of AR is a prerequisite for any effect of NE on these cells. We observed that both trauma and OA BMSCs, do express α2A-, α2B-, α2C-, and β2-AR, while α1A-, α1B-, α1D- β1- and β3-AR were not detected on day 0. This is in line with an earlier study in which α1- and β2-AR were identified immunohistochemically in BMSCs obtained from trauma patients [13]. In contrast, Han et al. could only confirm the expression of α1A-, α1B- and α1D-AR in rat BMSCs while α2- and β-AR were not detected [25]. One possible reason for this discrepancy might be that the AR gene expression profile differs between species [26] or that gene expression of resident BMSCs changes in the long-lasting pathophysiological microenvironment of OA damaged joint tissues [27]. This might also explain why the expression of α2C- and β2-AR was higher in trauma BMSCs compared to OA BMSCs. Furthermore, the concentration of NE in the synovial fluid of trauma patients is higher as in OA patients [13]. This difference in local NE concentration might influence the gene expression of diverse AR subtypes in a different manner [28,29]. To consider possible autocrine effects, we investigated gene expression of TH, the key enzyme of the catecholamine synthesis [30]. In line with earlier results, TH was neither detected in human trauma nor in OA BMSCs [13]. Thus, autocrine effects could be excluded. After 7 days in monolayer culture, α1A- and α1B-AR were up-regulated in OA BMSCs, while the expression of α2B-AR decreased. In contrast, AR expression profile of trauma BMSCs did not change during monolayer culture. This is in line with the fact that the gene expression of BMSCs might be more vulnerable in OA as a result of the pathophysiological microenvironment [27]. Besides local inflammation in the joint, also systemic inflammation is associated with the disease indicated by elevated serum interleukin levels [31]. In a recent study, we demonstrated that the treatment of articular chondrocytes with OA-characteristic low dose IL-1β resulted in the down-regulation of all AR subtypes already after 7 days [27]. Similarly, the pro-inflammatory microenvironment in the OA bone marrow may have primed the OA BMSCs leading to a stronger response compared to trauma BMSCs with respect to AR regulation in monolayer culture. Next, we investigated the proliferation capacity of human trauma and OA BMSCs under physioxia. We did not detect any differences between trauma and OA BMSCs indicating that the proliferation potential of trauma and OA BMSCs is not impaired, although OA donors were twice as old as trauma donors. This finding is in line with an earlier study demonstrating that donor age has only an influence on activation from dormancy and early proliferation, but does not affect BMSC proliferation after passage 1 in vitro [32]. Due to the increased NE concentration in the synovial fluid of trauma and knee OA patients we examined the influence of NE on the proliferation of trauma and OA BMSCs. We observed that the proliferation capacity of these cells is inhibited dose-dependently by treatment with NE for 7 days. Especially high concentrations of NE (10^−5^ to 10^−6^ M) decreased the cell count significantly in comparison to the untreated control group. Cytotoxicity expressed as LDH activity was not affected by any treatment indicating that decreased cell counts are due to reduced proliferation potential and not the result of cytotoxic effects. In contrast, Han et al. as well as Kido et al. demonstrated that the treatment of rat BMSCs with NE in high concentrations (10^−4^ to 10^−7^ M) lead to an increased proliferation [25,33]. One explanation for these discrepancies might be that they investigated the proliferation potential of healthy BMSCs of a different species. In contrast, we studied human BMSCs. In addition, our BMSCs were derived from trauma and OA bone marrow both representing a pathophysiological microenvironment due to inflammatory responses after trauma or during OA progression [34,35]. It might well be that the inflammatory milieu changes the AR signaling as recently demonstrated [36]. Furthermore, rat BMSC treatment was carried out for 0−24 h under hyperoxic conditions while we treated human BMSCs for seven days under physioxic conditions. On the one hand, different oxygen concentrations might contribute to these discrepant results because oxygen tension has an influence on the proliferation and differentiation capacity of MSCs [37]. Also different treatment durations indicate that short-time stimulation of BMSCS might result in enhanced proliferation while long-term stimulation has opposite effects as it was shown for other cell types [38]. Similarly, earlier studies demonstrated that NE induces cancer cell proliferation and that beta blockers are able to improve the clinical outcome of cancer [39,40,41]. This difference between somatic stem cells, such as our BMSCs, and cancer stem cells can be explained by the essential differences in their niche. The microenvironment of BMSCs provides proliferation inhibitory signals preventing tumorigenesis, while in cancer stem cells, the loss of this niche results in a different proliferative response [42].

To evaluate if the inhibitory effects of NE on the proliferation capacity are a result of AR regulation, we investigated the AR gene expression profile of OA and trauma BMSCs treated with NE. Treatment with NE in high concentrations (10^−6^ M) resulted in a slight but not significant α2A- and α2B-AR down-regulation in OA BMSCs, but especially β2-AR expression did not change after treatment with NE. In contrast, AR expression profile of trauma BMSCs was not affected by treatment with NE. On the one hand, these data indicate that the pathophysiological microenvironment of OA damaged joint tissues might result in the fact that MSCs are more affected by an imbalance of external factors such as growth factors, inflammatory mediators or hormones [9,10,11] compared to MSCs resident in healthy joint tissues. On the other hand, NE-induced inhibition of proliferation does not seem to be a result of α2-AR down-regulation, because a slight down-regulation of α2-AR has only been observed in OA BMSCs. Thus, we concluded that inhibitory effects of NE might be mediated by the β2-AR, because it seems to be the most stable AR being least affected by alterations of environmental factors.

After having observed significant effects of NE on the proliferation capacity, we aspired to identify the intracellular signaling pathways mediating those effects. We investigated the regulation of the major AR-coupled pathways, PKA and ERK1/2 [19,43]. Activation of these signaling pathways is related to the regulation of cell proliferation in different cell types [44,45]. We demonstrated that the treatment of trauma and OA BMSCs with NE results in PKA and ERK1/2 activation already after 5 min. Our data indicate that the major pathway for NE-mediated effects on proliferation of human trauma and OA BMSCs is the ERK1/2 pathway because phosphorylation of ERK1/2 is more pronounced than phosphorylation of PKA. Furthermore, phosphorylation of ERK1/2 remained stable for a longer period while PKA activation declined after 30 min. of stimulation in trauma BMSCs. In diverse cell types, short time activation of ERK1/2 leads to an increase of proliferation while chronic activation results in decreased proliferation rates [46,47]. Furthermore, this is also in line with the results of Kido et al. and Han et al. as mentioned above [25,33]: Short-time stimulation of BMSCs with NE results in increased proliferation rates possibly mediated via activation of ERK1/2. In contrast, long-time treatment of BMSCs with NE might lead to a decrease of proliferation as a result of chronic hyperactivation of ERK1/2 with subsequent cell cycle arrest.

The next step was to identify the AR subtype mediating the inhibitory effects of NE on the proliferation of human BMSCs. We demonstrated that the treatment with the specific β2-AR-agonist formoterol inhibited proliferation of trauma and OA BMSCs to the same extent as the treatment with NE in high concentrations, while the treatment with the specific α2-AR agonist UK14,304 had no significant influence. These results indicate that the effects of NE on proliferation of human BMSCs are mainly mediated via β2-AR. For confirmation, we also applied specific AR antagonists. The specific α1-antagonist doxazosin could neither abolish NE-mediated proliferation effects nor PKA and ERK1/2 phosphorylation. Treatment with the specific α2-AR-antagonist yohimbine did also not influence proliferation rates. α2-AR activation leads to PKA inhibition and ERK1/2 activation [16,19], which is consistent with the ability of the specific α2-AR-antagonist yohimbine to reverse NE-mediated ERK1/2 activation but not to abolish NE-induced PKA phosphorylation in our experiments. Only propranolol, a specific β2-AR-antagonist reversed NE-mediated effects on proliferation of trauma and OA BMSCs and also on the phosphorylation of PKA and ERK1/2. Thus, the NE-mediated effects are the result of β2-AR-mediated PKA and ERK1/2 activation. Using global ERK1/2 and PKA regulators we could show that modulation of these pathways can regulate proliferation. However, a clear link between proliferation and ERK1/2 phosphorylation in different human cell types was published in numerous previous studies [48,49,50,51]. Therefore, we focused on the effects of AR-modulators without manipulating other ERK1/2- or PKA-dependent processes in the cell.

In conclusion, we demonstrated that the proliferation capacity of BMSCs obtained from trauma and OA patients is inhibited in the presence of NE in physiological concentrations and that this effect results from β2-AR-mediated ERK1/2 and PKA activation with ERK1/2 being the more active pathway. Thus, we suggest that NE suppresses the regeneration capacity of articular cartilage by mainly inhibiting proliferation of BMSCs. This inhibitory effect of NE is not dependent on differences in the BMSCs microenvironment (trauma vs. OA). Therefore, targeting specific adrenergic receptors might represent a promising approach for the development of future therapeutic options for both trauma-induced or OA-related articular cartilage regeneration.

## 4. Materials and Methods

### 4.1. Patients

Human bone marrow was harvested from the iliac crests of patients after knee trauma and from OA patients undergoing total joint replacement. Knee trauma patients (crucial ligament rupture, meniscal tear) underwent surgery 2–3 weeks after injury upon the initial swelling and hematoma abated. The Ethics Committees of the University of Regensburg (vote number 13-101-0135) and of the Goethe University Frankfurt am Main (vote number 148-17B) approved the project. Experiments were performed in consideration of relevant guidelines and regulations. All patients provided their written consent after being informed about the purpose of the study. A total of 31 patients as BMSC donors were recruited for the study. Patient characteristics are shown in Table 1. We analyzed a random set of trauma and OA samples. There was only one exclusion criterion, namely that none of the donors received adrenoceptor-modulating drugs such as beta blockers. The mean age of trauma patients was significantly lower than the mean age of OA patients (*p* < 0.001).

### 4.2. Cell Culture

BMSCs were isolated from the bone marrow as described previously [52]. MSC separation from the bone marrow aspirate was performed by Ficoll density-gradient centrifugation. The cells were seeded in 75cm² tissue culture flasks in basic medium DMEM/F-12 (1:1) (Dubecco’s Modified Eagle Medium, Gibco^®®^ life technologies™, Thermo Fisher Scientific, Darmstadt, Germany), supplemented with 10% fetal bovine serum for mesenchymal stem cells (FBS MSC, PAN BIOTECH, Aldenbach, Germany) and 1% penicillin/ streptomycin (Gibco^®®^ life technologies™, Thermo Fisher Scientific, Darmstadt, Germany). Cells were cultured at 37 °C under physioxic conditions (2% O_2_, 5% CO_2_) [53], medium was changed twice per week. After expansion, cells were detached at 90–95% confluency by treatment with Accutase^®®^ solution (Sigma-Aldrich, Munich, Germany), counted manually and seeded for proliferation experiments as described below (see 4.3.). Stem cell-specific surface markers of MSCs were investigated in cells of randomly selected donors by standardized FACS analysis of CD73, CD90, and CD105 (positive markers) as well as HLA-DR, CD11b, CD19, CD34, and CD45 (negative markers) as suggested by The Mesenchymal and Tissue Stem Cell Committee of the International Society for Cellular Therapy and as described by us earlier [54,55,56]. Both trauma and OA BMSCs were positive for the surface markers CD73, CD90, and CD105 and were negative for HLA-DR, CD11b, CD19, CD34, and CD45 (Figure S3).

### 4.3. Proliferation Experiments

BMSCs were seeded at a density of 2.667 cells/cm² and allowed to adhere overnight at 37 °C under physioxic conditions (2% O_2_, 5% CO_2_). We used both trauma and OA BMSCS at passages 2–4. Medium was changed after 24 h and supplemented with NE ((±)-Norepinephrine (+)-bitartrate salt, Sigma-Aldrich, Munich, Germany) in different concentrations (10^−9^ to 10^−5^ M), the specific β_2_-AR agonist formoterol (10^−7^ M, formoterol hemifumerate, Tocris Bioscience, Bristol, UK) or the specific α_2_-AR-agonist UK14,304 (10^−8^ M, UK14,304 tartrate, Tocris Bioscience, Bristol, UK). To investigate if effects are reversible, cells were treated with NE (10^−6^ M) in combination with the specific α1-AR-antagonist doxazosin (10^−7^ M, doxazosin mesylate, Tocris Bioscience, Bristol, UK), the specific α2-AR-antagonist yohimbine (10^−7^ M, yohimbine hydrochloride, Tocris Bioscience, Bristol, UK) or the specific β-AR-antagonist propranolol (10^−6^ M, (S)-(−)-Proporanolol hydrochloride, Tocris Bioscience, Bristol, UK) for seven days in further experiments. Medium was replaced and freshly supplemented every two days. Untreated BMSCs served as control group, where the medium without any agonist or antagonist was replaced every two days and the total medium volume was adjusted to the total volume of the treated groups.

BMSC phenotype was investigated by 10x magnification (OLYMPUS CKX41, single replicate per donor). Cells were cultured for seven days and detached as described above. Total cell count and the number of living cells were determined and compared to an untreated control group. To investigate cell viability, supernatants were obtained on day 7 and used immediately for cell viability assays as described below.

### 4.4. Determination of Cell Viability

To demonstrate possible cytotoxic effects, cell viability was determined by measuring LDH activity in cell culture supernatants using the TaKaRa LDH Cytotoxicity Detection Kit MK401 (Takara, Kusatsu, Japan) according to the manufacturer’s instructions. Supernatants were harvested on day 7. In addition, culture medium without cells and dead control treated with 1% Triton-X-100 (Merck KGaA, Darmstadt, Germany) for 10 min were used as controls. Samples of each donor were analyzed in duplicate in a 96-well plate at 490 nm (TECAN infinite M200PRO, TECAN Magellan V 7.2 (c) 2016).

### 4.5. Western Blot Analysis

To identify AR-dependent signaling pathways cells were treated with NE 10^−6^ M for 5, 10, 30 and 60 min at 37 °C and physioxic conditions (2% O_2_, 5% CO_2_) and the phosphorylation of the two major AR-related pathways PKA and ERK1/2 was investigated (single replicate per donor). At least 6 × 10^4^ cells were placed in six well plates and allowed to adhere overnight. After 24 h medium was replaced to remove non-adherent cells. In addition to an untreated group, medium was supplemented with NE 10^−6^ M. In further experiments, NE 10^−6^ M was combined with 10^−7^ M doxazosin, 10^−7^ M yohimbine or 10^−6^ M propranolol. Cells were incubated for 10 min at 37 °C under physioxic conditions (2% O_2_, 5% CO_2_). Subsequently, wells were washed three times with DPBS (1X, Dulbecco’s Phosphate Buffered Saline, -CaCl_2_, -MgCl_2_, Gibco^®®^ life technologies™, Thermo Fisher Scientific, Darmstadt, Germany). Cells were harvested using PhosphoSafe™ Extraction Reagent according to the manufacturer’s instructions (Merck KGaA, Darmstadt, Germany). A 10% SDS-PAGE-gel was loaded with the samples and electrotransferred to a polyvinylidene difluoride membrane (Amersham™ Hybond™ P 0.45 PVDF Blotting Membrane, GE Healthcare Life science, Solingen, Germany). Membranes were blocked in 5% BSA for 1 h at room temperature. Afterwards membranes were incubated with primary antibodies for at least 16 h at 4 °C. Primary and secondary antibodies are listed in Table 2, antibodies were diluted as mentioned below. Incubation was stopped by washing with TBST, followed by incubation with HRP-conjugated secondary antibody for 1 h at room temperature. After washing with TBST, protein expression was visualized using an enhanced chemiluminescence (ECL) detection solution (Bio-Rad Laboratories) and detected with Molecular Imager^®^ ChemiDoc^TM^ XRS+ with Image Lab^TM^ Software (Version 5.2.1 build 11, © Bio-Rad Laboratories). Quantification was made with ImageJ 1.52a (Wayne Rasband, National Institute of Health, USA) with GAPDH as loading control.

### 4.6. RNA Isolation and cDNA Synthesis

RNA was isolated using NucleoSpin^®®^ RNA/ Protein kit (MACHEREY-NAGEL, Düren, Germany) according to the manufacturer’s instructions. RNA concentration was determined with NanoDrop One spectrophotometer. cDNA synthesis was performed using the qScript™ cDNA Synthesis Kit (Quanta Biosciences, VWR, Darmstadt, Germany) in a thermocycler qTOWER^3^ G (Analytik Jena AG, Jena, Germany) using qPCRsoft 3.4 (© 2009–2016 © by Analytik Jena AG, Jena, Germany). Samples were incubated at 22 °C for 5 min, at 42 °C for 30 min, at 85 °C for 5 min, followed by cooling at 4 °C for 60 min.

### 4.7. Real-Time RT-PCR and Gel Electrophoresis

To analyse gene expression of ARs real-time RT-PCR was performed using TaqPCR Master Mix Kit (QUIAGEN, Hilden, Germany) on day 0 and day 7 (duplicates per donor). 10 µL TaqPCR Master Mix, 5 µL RNAse free water, 2 µM of each primer and 10 ng cDNA were added to a final volume of 20 µL. *Human* primers were obtained by Thermo Fisher Custom Primers (Invitrogen by Thermo Fisher Scientific, Darmstadt, Germany), sequences are listed in Table 3. *Human GAPDH* served as housekeeping gene, *TH* gene expression was investigated to consider possible autocrine effects. PCR was performed in a thermocycler qTOWER^3^ G (Analytik Jena AG, Jena, Germany), programmed with qPCRsoft 3.4 (© 2009–2016 © by Analytik Jena AG, Jena, Germany) under following cycling conditions: at 94 °C for 0:01 min, followed by 36 cycles at 94 °C for 0:30 min, 64 °C for 0:30 min and 72 °C for 0:59 min. 

Samples were loaded onto a 1.8% agarose gel subsequently stained with GelRed Nucleic Acid Gel Stain (Biotium, Fremont, CA, USA). Bands were detected and quantified with Molecular Imager^®^ ChemiDoc^TM^ XRS+ with Image Lab^TM^ Software (Version 5.2.1 build 11, © Bio-Rad Laboratories). Bands were standardized to *GAPDH* used as endogenous control.

### 4.8. Statistical Analysis

Data were analyzed with SigmaPlot software (SigmaPlot V.13, Systat Software, Erkrath, Germany) via Kruskal-Wallis ANOVA on ranks using the post hoc tests Bonferroni or Mann-Whitney U-test. We used Mann-Whitney U-test to analyze the data between two independent groups. *p* values ≤ 0.05 were considered statistically significant. Experiments were performed with samples of 3–13 patients per experiment using *n* = 6–7 samples for proliferation experiments under NE influence, *n* = 4 samples for AR gene expression screenings, *n* = 4–7 samples for proliferation experiments under NE plus antagonist influence (due to clear and significant results no further replicates were analyzed), *n* = 2–4 samples for intracellular signaling analysis via Western blot (due to clear and significant results no further replicates were analyzed). Detailed information on replicates is given in the figure legends.

## Figures and Tables

**Table 1:** Characteristics of patients under study.

Patient Characteristics	Trauma BMSC Number/Mean/(%)/(Range)	OA BMSC Number/Mean/(%)/(Range)
total (number, %)	14 (100%)	17 (100%)
female/male (number, %)	2/9 * (18.18%/81.82%)	1 / 16 (5.88% / 94.12%)
age (years (mean ± stdd., (range)))	34.08 ± 10.79 (23–51)	66.44 ± 6.72 (52–76)

Abbreviations: stdd—standard deviation; * gender of three donors unknown.

**Table 2:** The antibodies used for Western Blot.

Antibody	Target Protein/Species/Antibody/Manufacture/Dilution
ERK	p44/42 MAPK (ERK1/2) (3A7)/mouse/mAb/Cell Signaling Technology^®®^/1:2500
p-ERK	p-p44/42 MAPK (T202/ Y204)/rabbit/mAb/Cell Signaling Technology^®®^/1:2500
PKA	ab32514, PKA R2, (Y116), 0,15 mg/mL/rabbit/mAb/Abcam/ 1:5000
p-PKA	ab32390, PKA R2 (E151) (phospho S99), 0,351 mg/mL/rabbit/ mAb/Abcam/1:2500
GAPDH	GAPDH Loading control antibody (GA1R)/mouse/mAb/ invitrogen by Thermo Fisher Scientific/1:2500
anti-mouse	Polyclonal Rabbit Anti-Mouse Immunglobulins/HRP/Dako, Glostrup, Denmark/1:1000
anti-rabbit	Polyclonal Swine Anti-Rabbit Immunglobulins/HRP/Dako, Glostrup, Denmark/1:1000

**Table 3:** Primer sequences used for PCR (for *human* species).

Gene	NCBI Reference	Forward Primer (5′-3′)	Reverse Primer (5′-3′)
*GAPDH*	NM_001289745.2	CTC CTG TTC GAC AGT CAG CC	TTC CCG TTC TCA GCC TTG AC
*ADRA1A*	NM_000680.3	CCA TGC TCC AGC CAA GAG TT	TCC TGT CCT AGA CTT CCT CCC
*ADRA1B*	NM_000679.3	GTC CAC CGT CAT CTC CAT CG	GAA CAA GGA GCC AAG CGG TAG
*ADRA1D*	NM_000678.3	TGA CTT TCC GCG ATC TCC TG	TTA CCT GCC ACG GCC ATA AG
*ADRA2A*	NM_000681.3	TGG TCA TCG GAG TGT TCG TG	GCC CAC TAG GAA GAT GGC TC
*ADRA2B*	NM_000682.6	GAC ATT TCA CCG GCA ACA CC	GGG ACT GAG AAC CAG GAA GC
*ADRA2C*	NM_000683.3	CGA TGT GCT GTT TTG CAC CT	GGA TGT ACC AGG TCT CGT CG
*ADRB1*	NM_000684.2	TAG CAG GTG AAC TCG AAG CC	ATC TTC CAC TCC GGT CCT CT
*ADRB2*	NM_000024.5	CAG AGC CTG CTG ACC AAG AA	GCC TAA CGT CTT GAG GGC TT
*ADRB3*	NM_000025.2	GCC AAT TCT GCC TTCAAC CC	GCC AGA GGT TTT CCA CAG GT
*TH*	NM_000360.3	CAG GCA GAG GCC ATC ATG T	GTG GTC CAA GTC CAG GTC AG

## Supplementary Materials

Supplementary materials can be found at https://www.mdpi.com/1422-0067/21/11/3924/s1. Figure S1: LDH release of trauma and OA BMSCs after 7 days of treatment with NE alone and in combination with AR antagonists in monolayer culture n = 3–7). Figure S2: Effect of AR agonists on the proliferation and viability of trauma and OA BMSCs. Figure S3: Characterization of MSC-specific surface markers of BMSCs isolated from trauma and OA donors.

## Abbreviations

AR	Adrenergic receptor
BMSC cAMP	Bone marrow-derived stem cells Cyclic adenosine monophosphate
cDNA	Complementary desoxy ribonucleic acid
DMEM/F12	Dulbecco’s modified eagle’s medium and Ham’s F-12 Medium
DPBS	Dulbecco’s phosphate buffered saline
ECL	Enhanced chemiluminescence
ERK	Extracellular signal-regulated kinases
FBS	Fetal bovine serum
GAPDH	Glyceraldehyde-3-phosphate dehydrogenase
HRP IL-1β LDH	Horse radish peroxidase Interleukin-1β Lactate dehydrogenase
mRNA	Messenger ribonucleic acid
MSC	Mesenchymal stem cells
NE	Norepinephrine
OA	Osteoarthritis
P/S	Penicillin streptomycin
PCR	Polymerase chain reaction
pERK	Phosphorylated extracellular signal-regulated kinases
PKA	Protein kinase A
pPKA	Phosphorylated protein kinase A
PVDF	Polyvinylidenfluorid
RT-PCR	Reverse-transcriptase PCR
SDS-PAGE	Sodium dodecyl sulfate polyacrylamide gel electrophoresis
TBST	Tris-buffered saline with Tween20
TH	Tyrosine hydroxylase
