# Supplementary material for: Norepinephrine Inhibits the Proliferation of Human Bone Marrow-Derived Mesenchymal Stem Cells via β2-Adrenoceptor-Mediated ERK1/2 and PKA Phosphorylation
Source: Int J Mol Sci. 2020 May 30;21(11):3924. doi: 10.3390/ijms21113924 (PMC7312191; doi:10.3390/ijms21113924)
Supplement: Supplementary file 1 [file ijms-21-03924-s001.pdf]

Supplementary Material

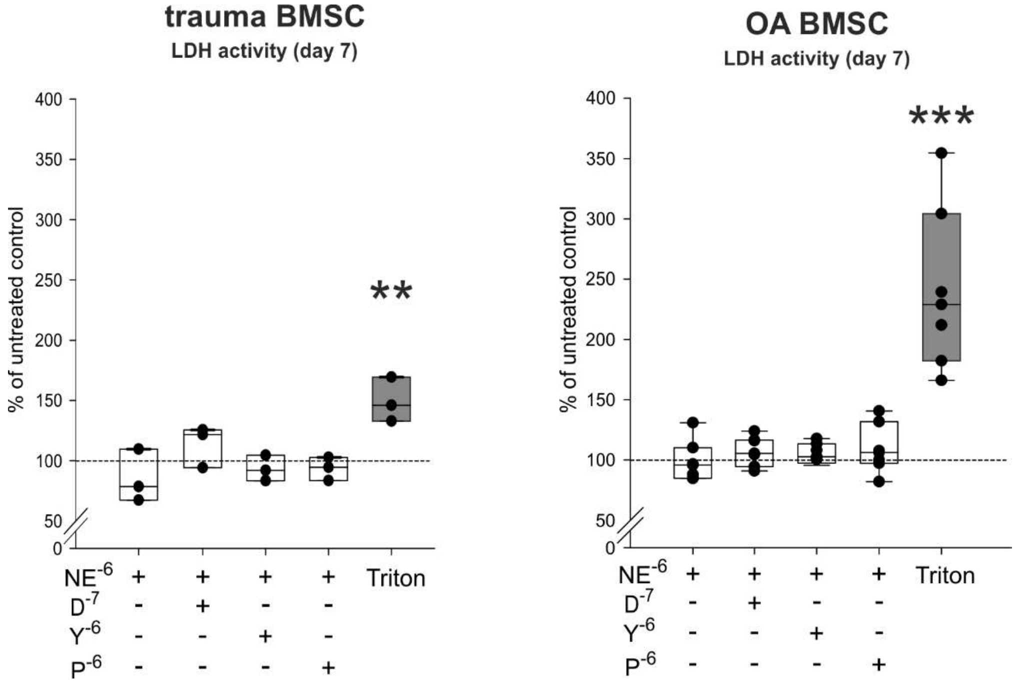

Supplementary Figure 1. LDH release of trauma and OA BMSCs after 7 days of treatment with NE alone and in combination with AR antagonists in monolayer culture n=3-7). Data are presented as box plots as described in the legend to figure 1 and as percent of the untreated control (control= 100 %, broken line). Significant p values are presented as **p≤0.01 or p≤0.001 to the untreated control.
Abbreviations: D-7 – doxazosin (α2-AR antagonist); Y-6 – yohimbine (α2-AR antagonist); P-6 – propranolol (β2-AR-antagonist)

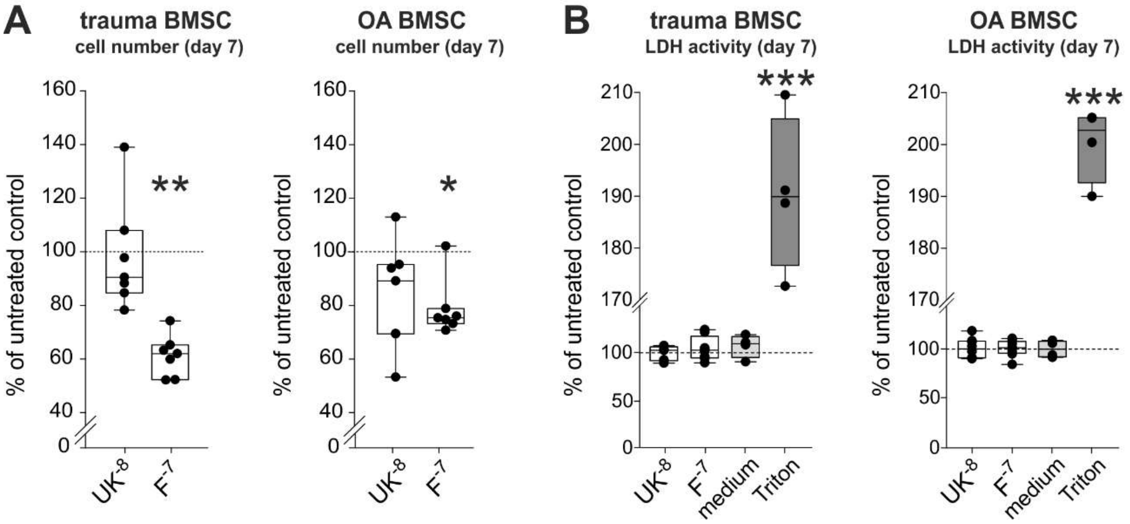

Supplementary Figure 2. Effect of AR agonists on the proliferation and viability of trauma and OA BMSCs (A) Vital cell count of trauma and OA BMSCs 7 days after treatment with α2-AR-agonist UK14,304 (UK, 10^{-8} M) and β2-AR-agonist formoterol (F, 10^{-7} M) (trauma BMSCs: p<0.001 for F 10^{-7} M vs. untreated control, OA BMSCs: p=0.026 for F 10^{-7} M vs. untreated control; n=7). (B) LDH release of trauma and OA BMSCs after treatment with α2-AR-agonist UK14,304 (UK, 10^{-8} M) and β2-AR-agonist formoterol (F, 10^{-7} M) (n=4). Data are presented as box plots as described in the legend to Figure 1 and as percent of the untreated control (control= 100 %, broken line). Significant p values are presented as *p≤0.05, **p≤0.01 and ***p<0.001 to the untreated control.

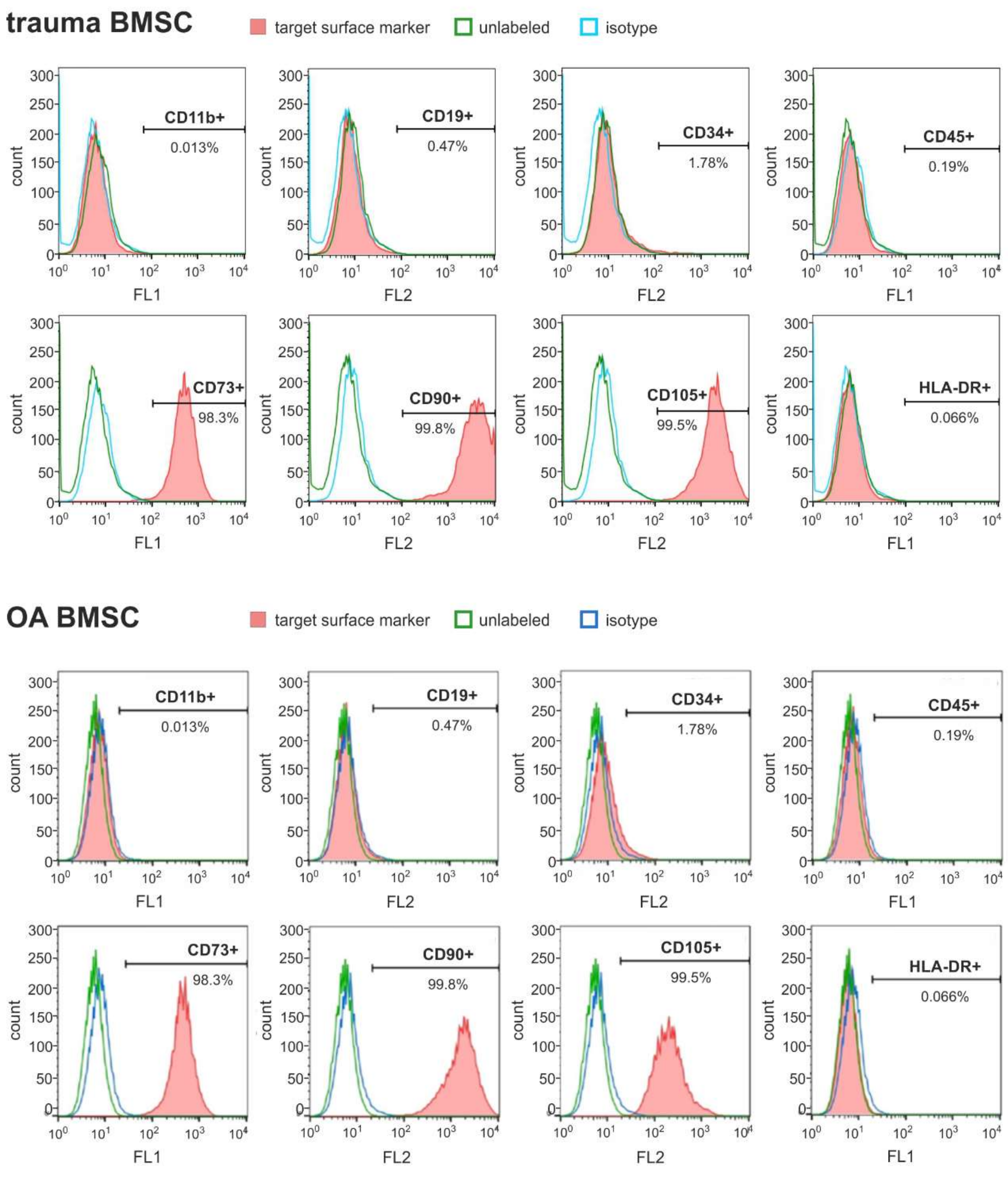

Supplementary Figure 3. Characterization of MSC-specific surface markers of BMSCs isolated from trauma and OA donors. BMSCs of both donor groups were negative for CD11b, CD19, CD34, CD45, and HLA-DR and were positive for CD73, CD90, and CD105 as suggested by *The Mesenchymal and Tissue Stem Cell Committee of the International Society for Cellular Therapy* (representative FACS analysis pictures of n=1 trauma and n=1 OA BMSCs).
